# A Practical Perspective: The Effect of Ligand Conformers on the Negative Image-Based Screening

**DOI:** 10.3390/ijms20112779

**Published:** 2019-06-06

**Authors:** Mira Ahinko, Sami T. Kurkinen, Sanna P. Niinivehmas, Olli T. Pentikäinen, Pekka A. Postila

**Affiliations:** 1 Department of Biological and Environmental Science & Nanoscience Center, University of Jyvaskyla, P.O. Box 35, FI-40014 Jyvaskyla, Finland; mira.m.k.ahinko@jyu.fi (M.A.); sami.t.kurkinen@utu.fi (S.T.K.); sanna.niinivehmas@utu.fi (S.P.N.); olli.pentikainen@utu.fi (O.T.P.); 2 Integrative Physiology and Pharmacy, Institute of Biomedicine, University of Turku, FI-20014 Turku, Finland; 3 Aurlide Ltd., FI-21420 Lieto, Finland

**Keywords:** negative image-based (NIB) screening, negative image-based rescoring (R-NiB), molecular docking, rigid docking, docking rescoring, virtual screening, structure-based drug discovery, cyclooxygenase-2 (COX-2)

## Abstract

Negative image-based (NIB) screening is a rigid molecular docking methodology that can also be employed in docking rescoring. During the NIB screening, a negative image is generated based on the target protein’s ligand-binding cavity by inverting its shape and electrostatics. The resulting NIB model is a drug-like entity or pseudo-ligand that is compared directly against ligand 3D conformers, as is done with a template compound in the ligand-based screening. This cavity-based rigid docking has been demonstrated to work with genuine drug targets in both benchmark testing and drug candidate/lead discovery. Firstly, the study explores in-depth the applicability of different ligand 3D conformer generation software for acquiring the best NIB screening results using cyclooxygenase-2 (COX-2) as the example system. Secondly, the entire NIB workflow from the protein structure preparation, model build-up, and ligand conformer generation to the similarity comparison is performed for COX-2. Accordingly, hands-on instructions are provided on how to employ the NIB methodology from start to finish, both with the rigid docking and docking rescoring using noncommercial software. The practical aspects of the NIB methodology, especially the effect of ligand conformers, are discussed thoroughly, thus, making the methodology accessible for new users.

## 1. Introduction

**Figure 1:**
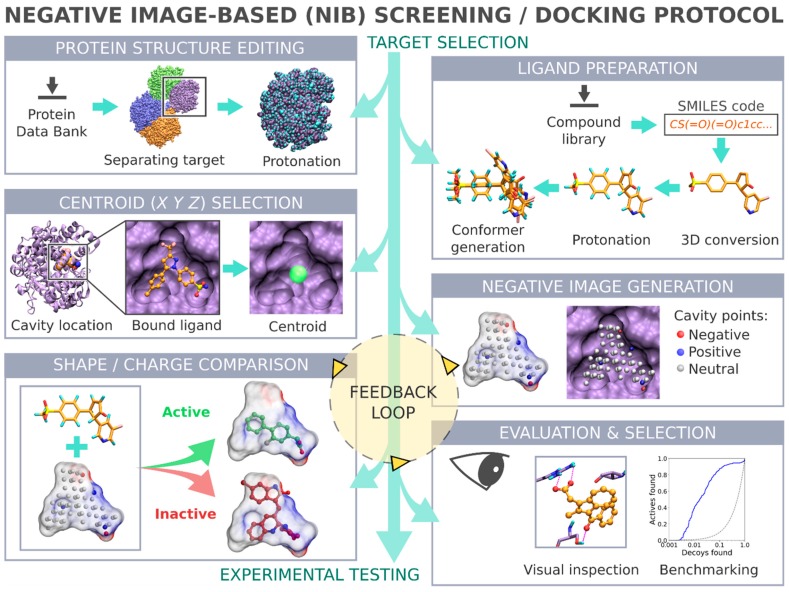
Negative image-based screening. The steps of a negative image-based (NIB) [1] screening or cavity-based rigid docking, which is presented using cyclooxygenase-2 (COX-2; Protein Data Bank (PDB): 3LN1 [2]; A chain) as a model system, include ligand preparation, protein 3D structure editing, cavity centroid (*X Y Z*) selection, negative image or NIB (negative image-based) model generation with PANTHER [3], geometry optimization or rigid docking with shape/charge comparison using ShaEP [4], visual evaluation of the highest scored ligand poses against the protein structure (e.g., BODIL [5]), and potential benchmark testing with the known ligand sets (e.g., ROCKER [6]) before the virtual screening against a commercial compound database, compound selection, and in vitro testing. In the receiver operating characteristics (ROC) plot, the blue line designates the NIB enrichment, and the dashed line outlines the random selection with the area under curve (AUC) value of 0.50.

Negative image-based (NIB) screening (Figure 1) is a rigid molecular docking methodology that combines the key strengths of both the structure- and ligand-based computer-aided drug discovery approaches [1]. The NIB relies primarily on the 3D coordinates of the target protein’s structure, especially its ligand-binding cavity (Figure 1), and the geometry optimization (or rigid docking) is performed similarly to the traditional ligand-based screening.

In the NIB screening, a negative image is built based on the target protein’s ligand-binding cavity shape and electrostatics (Figure 1). The NIB model ideally encompasses those key shape features of the target’s cavity required for the potent ligand binding. The NIB model generation, which is done using the cavity detection software PANTHER [3], takes into account explicit water molecules, cofactors and ions, user-defined restrictions, and alternative residue protonation. A NIB model can be built based solely on protein 3D structure information (Figure 1) and, thus, without prior knowledge on target-specific active and inactive ligands. The resulting NIB model functions as a template or pseudo-ligand directly in the shape/electrostatics similarity comparison against ligand 3D conformers included in the screening compound libraries. The ligand preparation and similarity comparison against the model (Figure 1) is done using established ligand-based screening tools [4,7].

Whereas standard flexible docking relies on estimating the favorability of ligand-receptor complexes by summing up the weak interactions, such as hydrogen bonding and the hydrophobic effect, the NIB focuses squarely on the shape/electrostatics similarity of the molecular recognition process. Despite the apparent simplicity of this shape-centric approach, the benchmarking has shown thatthe NIB produces high enrichment as indicated by the area under curve (AUC) values and early enrichment factors with various targets [1,3,8]. The methodology is especially suitable for the targets with well-defined cavities such as nuclear receptors, but, in practice, even a sub-cavity or a shallow groove can be used to build an effective negative image. As such, the NIB has been used to assist the structure-activity relationship analysis of the 3-phenylcoumarin analog series with the 17-hydroxysteroid dehydrogenase 1 [9], monoamine oxidase B [10], and UDP-glucuronosyltransferase 1A10 [11], as well as to facilitate the discovery of novel estrogen receptor α ligands [12] and retinoic acid-related orphan receptor γ(t) inverse agonists [13].

Applying 3D similarity- or shape-based methods in the virtual screening schemes increases the diversity of the discovered compounds [14]. With the NIB, the docked ligand and protein can overlap somewhat, and, while this can weaken the compound’s ranking, no ligands are skipped entirely due to the clashes as can happen with the flexible docking algorithms. The upside of tolerating the overlaps is that those novel scaffolds or functional moieties producing a good partial match with the target’s cavity are readily put forward. This is advantageous because docking can put forth not only new compounds but also functional fragments to be incorporated into novel drug constructs via organic synthesis [15]. Moreover, Molecular Mechanics/Generalized Born and Surface Area (MM/GBSA) calculations, for example, can be performed to optimize the rigid docking poses inside the target protein’s cavity for improving the NIB enrichment [8].

**Figure 2:**
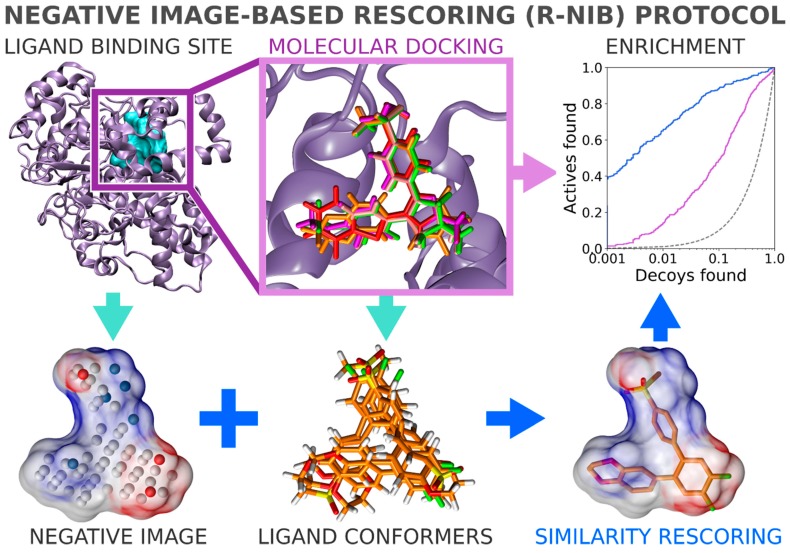
Negative image-based rescoring. The negative image-based rescoring (R-NiB) [16] begins with the flexible docking of ligands (green/red/magenta/orange stick models) into the binding site (magenta box) of cyclooxygenase-2 (COX-2; magenta cartoon; PDB: 3LN1 [2]; A chain) using a flexible molecular docking algorithm (e.g., PLANTS [17]). Here, the centroid coordinates of the bound inhibitor celecoxib (cyan opaque surface) are used in the docking. Several alternative flexible docking poses (e.g., *n* = 10) are output for the rescoring phase. Next, a cavity-based NIB model is generated with PANTHER [3] using the same celecoxib-based cavity centroid that was used in the original docking. The shape/electrostatics of the NIB model are directly compared against the ligand 3D conformers without geometry optimization using ShaEP [4]. With the directory of useful decoys (DUD) set [18], the initial docking enrichment (magenta line), which is already well above the random limit (dotted line), is improved by the R-NiB treatment (blue line). See Figure 1 for interpretation.

In general, flexible docking is better positioned to sample the possible ligand poses than the rigid docking approach. Therefore, the NIB methodology was recently repurposed for rescoring existing molecular docking solutions [16]. The NIB rescoring (R-NiB; Figure 2) of explicit docking poses was shown to improve the docking performance markedly, especially the very early enrichment, with several targets. This includes cyclooxygenase-2 (COX-2; enzyme commission number 1.14.99.1; Figure 1 and Figure 2), which catalyzes the conversion of arachidonic acid to prostaglandin endoperoxide H2 and was used as a NIB screening and docking rescoring example in this study. In short, the NIB is not only a powerful docking technique (Figure 1), but it is also a docking rescoring (Figure 2) methodology that has the potential for wide-scale application.

The study provides simple step-by-step instructions on how to perform rigid docking (Figure 1) or docking rescoring (Figure 2) using the NIB methodology with non-commercial software. The in-depth examination of the settings together with discussion on the notable exceptions is outlined using practical COX-2 screening examples (Figure 1 and Figure 2). Furthermore, several popular ligand 3D conformer generation algorithms are tested with the COX-2 test sets and compared to outline the optimal scheme for the rigid docking with the NIB methodology.

## 2. Results

The negative image-based (NIB; Figure 1) screening [1,3,8] and the negative image-based rescoring (R-NiB; Figure 2) [16] protocols are presented below as stepwise workflows.

The practical aspects of the NIB and R-NiB methodologies are discussed below using a virtual screening or benchmarking example, i.e., the screening is performed using the directory of useful decoys (DUD) test set [18,19] and a celecoxib-bound cyclooxygenase-2 (COX-2) protein 3D structure (Figure 1 and Figure 2; Protein Data Bank (PDB): 3LN1 [2]). Note that the NIB protocol (commands #1–23) is executed in the BASH command line interface (or terminal) in the UNIX/LINUX environment. Furthermore, three alternative conformer generators (Table 1) were tested for the NIB in addition to OBABEL, which is used in the benchmarking example. Finally, the R-NiB is performed using the flexible docking poses generated by PLANTS to improve the enrichment. The rescoring relies either solely on the ShaEP-based complementarity or similarity scoring (commands #24–35) or the combined and re-weighted PLANTS- and ShaEP-based consensus scoring (commands #36–41).

**Figure 3:**
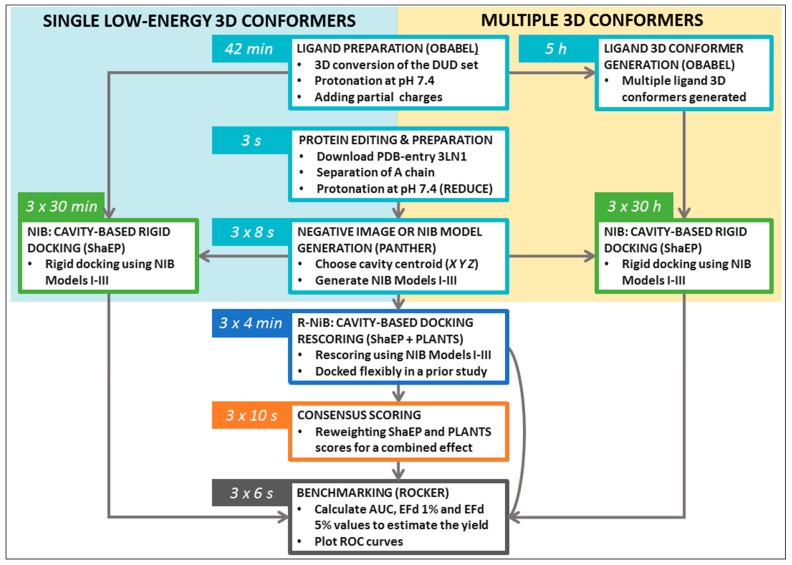
The duration of the protocol steps for the benchmarking example. The negative image-based (NIB; Figure 1) [1] screening or rigid docking can be done either using single low-energy conformers (Table 2) or using multiple conformers (Table 3). Going through the negative image-based rescoring (R-NiB; Figure 2) [16] protocol takes considerably less time (Table 4) because it is done using explicit PLANTS docking poses taken from a prior study [16]. Moreover, the rescoring process does not require geometry optimization in addition to the shape/charge similarity comparison. The execution of the NIB and R-NiB protocols with the cyclooxygenase-2 (COX-2) DUD test set can take less or more time depending on the used computer set-up. For simplicity, all the steps in the workflow are done using a single processor, but the process, especially the NIB screening with multiple ligand 3D conformers, can be sped up substantially by dividing the ligand sets into separate batches that are processed separately.

The terminal commands and further practical information are given in the Supplementary Material (README.txt, commands.txt) to assist the execution of trial runs of the protocols. The NIB protocol testing using the single low-energy conformers (Table 2) takes ~10-fold less time than with the multiple conformers (Table 3); furthermore, the R-NiB testing (Table 4) is substantially faster than the rigid docking, because the flexible docking poses are provided premade, and no geometry optimization is performed with ShaEP (Figure 3). Though the specific commands are not given to avoid repetition, the protocols were also tested using the DUD-E test set and the PDB-entry 1CX2 [20].

### 2.1. Ligand Preparation: 3D Conversion, Protonation, and Partial Charges

In the NIB screening (Figure 1), the rigidly docked ligand 3D conformers are generated ab initio with a separate software (Table 1); however, depending on the target protein and the ligand sets one can acquire high enrichment using only a few or even a single low-energy conformer. Before performing the cavity-based rigid docking with a single conformer or multiple conformers, the 3D coordinates (simplified molecular-input line-entry system (SMILES)-to-MOL2), partial charges and ionization/protonation states of the small-molecules need to be generated (Figure 1). This is achieved using, for example, LIGPREP in MAESTRO or MARVIN, but non-commercial software such as RDKit or OBABEL [21] can also be used. It is crucial that the pH is set to match the conditions of the activity assay (e.g., pH 7.4) during the protonation.

The DUD [19] ligands for COX-2 were converted from the SMILES format into the MOL2 format using OBABEL [21] (command #1). A single 3D conformer was generated for each ligand included in the set. Next, the protonation of the ligands was set to match pH 7.4 (command #2), and the partial charges were inserted using the Merck Molecular Force Field 94 (MMFF94) [22] (command #3) with OBABEL [21]. For comparison, the ligands were also prepped using LIGPREP in MAESTRO, MARVIN, and RDKit (Table 1). With COX-2, the NIB screening produces high enrichment directly using these single low-energy 3D conformers, and, for this reason, one can choose to skip the 3D conformer generation step to save time when going through the protocol (Figure 3).

### 2.2. Ligand Preparation: 3D Conformer Generation

Ultra-fast speed and computational efficiency are hallmarks of both the NIB screening (Figure 1) and the ligand-based screening [1,3,8]. This is largely because the different ligand 3D conformers are not sampled on the fly against the protein 3D structure during the rigid docking, as is done in the flexible molecular docking. Instead, several low-energy conformers are generated for each ligand prior to the eventual similarity screening and geometry optimization with the cavity-based NIB model (Figure 1). The ligand 3D conformer generation can be done using either non-commercial or commercial software tools with varying results (Table 1). The conformer generation, as well as the eventual cavity-based rigid docking using the multiple conformers, is a lot more time consuming than performing the NIB screening with single low-energy conformers (Figure 3). Alas, one should not expect that single conformers would work in all screening experiments, although this is the case with the COX-2 benchmarking.

The protonated ligand 3D coordinates were used as an input to generate multiple conformers using the --confab option in OBABEL [21] (command #4). By default, an extensive number of conformers is generated, and, to avoid this, the output was limited with two basic options: The maximum number of conformers (-conf; from 1,000,000 to 100,000) and the root mean square deviation cutoff (--rcutoff; from 0.1 to 1.0). For comparison, the ligand 3D conformer generation was also done using other conformer generators (Table 1).

### 2.3. Selecting the Target Protein 3D Structure

**Figure 4:**
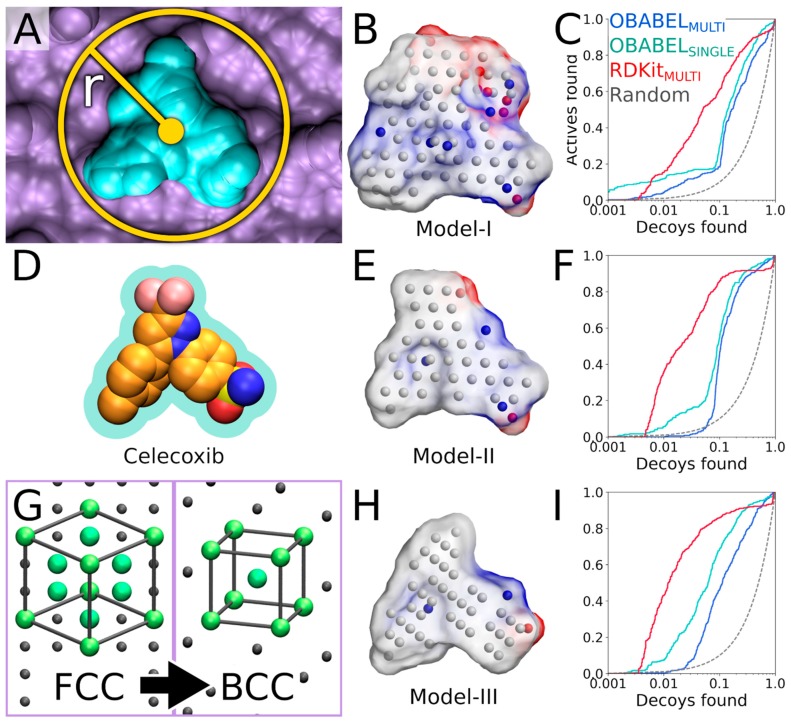
The negative image-based screening benchmarking evolution. (**A**) A cross section of the ligand-binding cavity (cyan) of the cyclooxygenase-2 (magenta; PDB: 3LN1; A chain) shown with the cavity centroid and detection radius (r = 10 Å). (**B**) The NIB (negative image-based) Model I, generated using the default PANTHER settings, (**C**) produced higher enrichment for the DUD test set using RDKit multi-conformer set (red line) than the multi-conformer OBABEL set (blue line; Table 3). The single-conformer OBABEL set (cyan line) resulted in higher early enrichment (Table 2) than its multi-conformer set. (**D**) The bound inhibitor (CPK model) is shown with the extra 1.5 Å volume. (**E**) Model II, fashioned using the 1.5 Å ligand distance limit, has roughly similar shape as the inhibitor (**D** versus **E**). (**F**) The enrichment was improved with Model II for the RDKit set over the prior model; however, the early enrichment weakened with both OBABEL sets (Table 2 and Table 3). (**G**) Models I and II were generated using the face-centered cubic (FCC) packing. The body-centered cubic (BCC) lattice packing was used for Model III. (**H**) Model III has less dense packing than Model II (**E** versus **H**). (**I**) Model III worked best with the RDKit conformers, but the effect was lesser for the OBABEL sets (Table 2 and Table 3). See Figure 1 for interpretation.

The success of the NIB screening is dependent on the input protein 3D structure, especially its ligand-binding cavity conformation, used as a template for the negative image generation (Figure 1). The input structure selection follows the basic criteria that apply to standard molecular docking as well: The resolution should be sufficiently high, and the protein conformation should be able to accommodate the binding ligand. In principle, the PDB-entry does not have to house any known active compounds prior to the model generation, but a bound ligand can affect the cavity geometry via induced-fit effects. If included, the bound ligand(s) can assist in the NIB model generation by providing centroid coordinates (Figure 1A and Figure 4A), and they can assist in limiting the model scope to the known binding area. In some cases, using multiple protein structures in the model generation originating, for example, from molecular dynamics (MD) simulation trajectory can improve the NIB screening yield [1].

**Figure 5:**
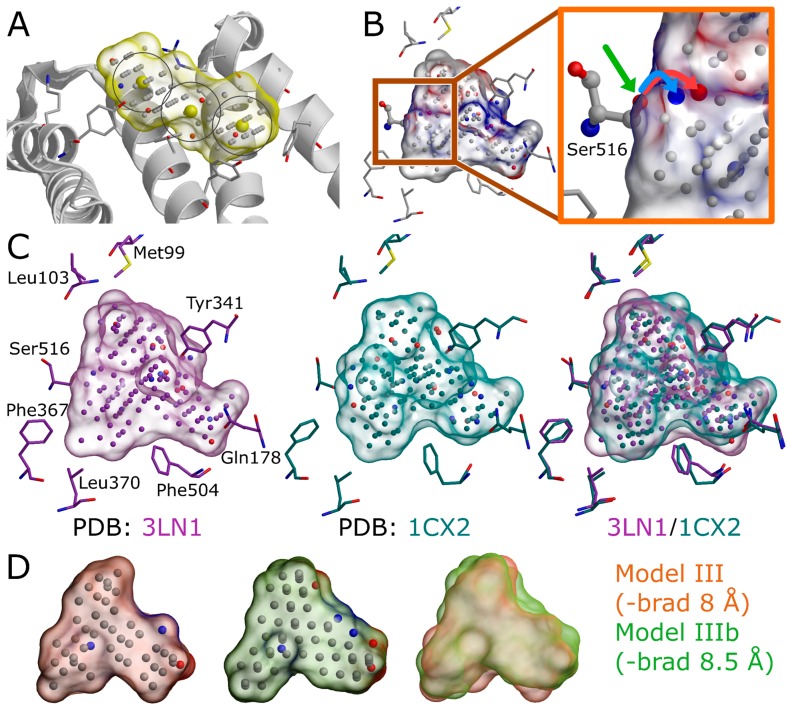
Valuable settings in the negative image generation. (**A**) A negative image or NIB (negative image-based) model (yellow surface) is built based on a shallow surface groove (white cartoon; PDB: 4BTB) [23] using three center (-center) coordinates (yellow spheres) and the multibox (-mbox) option in PANTHER [3]. (**B**) The effect of protonation for the model composition is shown with the hydroxyl group of Ser516 (ball-and-stick model) of cyclooxygenase-2 (PDB: 3LN1) [2]. If no specific protonation is given, two alternative angles of the hydroxyl’s polar proton (polar oxygen indicated with green arrow) are considered, and, thus, two models are generated where the mirroring cavity point is either positive (H-bond acceptor; red sphere) or negative (H-bond donor; blue sphere). The opposite charge pair is highlighted by cyan and red arrows in the close-up (orange box). (**C**) The input coordinates affect the resulting models as demonstrated by two PDB-entries 3LN1 [2] (purple surface) and 1CX2 [20] (turquoise surface). (**D**) The detection radius has a substantial effect, as highlighted by the model overlay. Model III (orange surface) is generated using otherwise similarly as Model IIIb (green surface), but the box radius (-brad) of 8 Å is increased to 8.5 Å. A few residues are shown as sticks for reference. See Figure 1 for interpretation.

Two PDB-entries were selected for the NIB screening with COX-2. The PDB-entry 3LN1 [2] (Figure 1, Figure 2 and Figure 5C) is used in the practical example; meanwhile, the PDB-entry 1CX2 [20] (Figure 5C) is used as an alternative input for which the applied commands are not shown due to their redundancy. The protein X-ray crystal structure was downloaded directly from the PDB in the terminal (command #5), but it can also be downloaded manually online (e.g., https://www.rcsb.org/).

### 2.4. Protein 3D Structure Editing and Preparation

The extra chains and other non-peptidic residues do not necessarily have to be removed for building NIB models using PANTHER [3], although their removal can make the process marginally faster. Even though the NIB model generation can be performed without protons added to the protein 3D structure, this can lead to several alternative cavity-based models. This is because certain residues can have alternative protonation states or bond angles for the protons that are responsible for the H-bonding (Figure 5B) and, thus, depend on the local environment. In fact, one should be mindful on how the added protons affect the charge distribution of the negative image and the eventual docking results. The protons can be added for the target structure and even its cofactors using external software (e.g., REDUCE [24]), in which case the alternative proton shuffling is omitted during the NIB model generation (Figure 1). The case-specific protonation of, for example, histidine and aspartic acid residues at the ligand-binding cavity, can be tricky, and, in unclear cases, one should employ protonation prediction algorithms such as PROPKA [25,26].

The A chain of the PDB-entry 3LN1 [2] was selected for the NIB model generation and, for improved computing efficiency, extracted into a separate PDB file (command #6; Figure 1) where the explicit protons were inserted using the default settings of REDUCE [24] (command #7; Figure 1). With COX-2, the outputted bond angles of the protons were visually assessed to be reasonable in BODIL [5]. Such an evaluation is necessary because, for example, the proton in the hydroxyl group of Ser516 side chain in the COX-2 active site could have an alternative angle that affects the resulting cavity point composition (Figure 5B).

### 2.5. Defining the Ligand-Binding Cavity Centroid

The NIB model generation using PANTHER [3] requires that the ligand-binding cavity location is designated beforehand (Figure 1A and Figure 4A). In other words, the user needs to have a concrete idea where the ligand binding should happen to focus on a specific location inside or on the surface of the protein. For this purpose, cavity detection software such as SITEMAP [27,28] or POVME 3.0 [29] can estimate the druggability and dimensions of protein cavities. In any case, the best scenario is to begin the NIB model generation with PANTHER using the centroid coordinates of a bound ligand already included in the PDB-entry. If not applicable, the cavity detection can begin from any arbitrary coordinate point given by the user (-center(s)) or by using any residue atom coordinate present near the cavity center (-basic multipoint). Overall, the centroid selection process is highly similar to choosing the center of radius for any standard docking routine.

The COX-2 inhibitor ligand celecoxib, or CEL, (residue 682 in the A chain; Figure 1 and Figure 4D), which is bound at the active site in the PDB-entry 3LN1 [2], was selected to define the cavity center (A-682) during the NIB model generation with PANTHER [3] (Figure 4A,D).

### 2.6. Generating a Negative Image of the Enzyme’s Ligand-Binding Cavity

The first NIB model is typically generated using the default settings in PANTHER [3] (Figure 1), after which it is critically evaluated, and, if needed, the settings are further tweaked. For convenience, especially at the later stages of the NIB model generation, the necessary settings are inserted directly into a PANTHER [3] input file using a text editor instead of typing and executing them in the terminal, as is done in the example below.

Firstly, the default PANTHER [3] input file (default.in) is generated (command #8). Secondly, PANTHER [3] is used to generate a preliminary NIB model (command #9) in the MOL2 format (Model I in Figure 4B). If the input protein would lack protons (command #*7*, not executed), altogether 12 alternative NIB models would be outputted for COX-2. This is because the cavity houses several residues capable of H-bonding, and each proton of the H-bond donor groups, such as the hydroxyl group, is given an alternative position that affects the charge distribution of the model (Figure 5B).

### 2.7. Estimating the Negative Image Viability and Tweaking the Settings

There are at least three major concerns regarding the model viability in the NIB screening:

(1) The NIB model must be restricted to the area of the cavity that facilitates the ligand binding.
(2) The NIB model shape should resemble, however loosely, an envisioned or actual ligand molecule occupying the cavity.
(3) The NIB model cavity points must contain crucial charge/electrostatics information required for mimicking the ligand-receptor H-bonding, as the shape similarity alone might not be enough for ensuring rigid docking success (Figure 5B).

The preliminary model or Model I (command #9) outputted by PANTHER [3] matches the cavity shape (Figure 4A versus 4B). It is composed of neutral filler atoms (grey dots in Figure 4B) and negatively or positively charged cavity points (red or blue dots, respectively, in Figure 4B) that have the opposite charges in comparison to the protein residues lining the cavity. The partial charges of protein (and possible cofactor) atoms must be pre-defined in a separate file, which contain by default AMBER (Assisted Model Building with Energy Refinement) force field-based charges (a PANTHER library charge.lib). In other words, the charge points face directly those residues capable of either accepting or donating protons in the H-bonds (Figure 5B). Before performing the similarity comparison with Model I (Figure 4B), a second model or Model II (Figure 4E) is generated using a 1.5 Å ligand distance limit (-ldlim) in PANTHER [3] (command #10). Though the two outputted models are roughly similar, Model II does not expand far away from the space taken by the inhibitor bound at the ligand-binding site in the input PDB-entry (Figure 4D versus 4E). In comparison, Model I, which was generated without the ligand distance limit but only relies on the 8 Å cavity detection radius, is visibly bulkier than Model II (Figure 4B versus 4E). This demonstrates that the ligand is loosely bound to the protein, the ligand does not fill the entire space available for binding, or the PANTHER parameterization for the NIB model generation is not optimal.

### 2.8. Rigid Docking by Aligning Ligands Against the Negative Image

The NIB screening is performed using the similarity comparison algorithm ShaEP [4] (Figure 1), which was originally developed for the ligand-based screening. The ligand 3D conformers are geometry optimized or rigidly docked based on the shape/electrostatics against the cavity-based NIB model (Figure 1). ShaEP [4] provides a similarity score from 1 to 0 for ranking the compounds from the best to the worst matches against the cavity-based model. Those ligand conformers matching the NIB model best are given the highest score, indicating the highest degree of similarity with the cavity space available for the ligand binding. If the model encompasses the key shape and charge features needed for the ligand binding, in theory, the NIB screening should put forward the best-matching molecules. While the charge can be an important factor for assuring correct H-bonding interactions in the rigid docking (Figure 4B), the shape is frequently the defining factor in the ligand-receptor complex formation.

The rigid docking is performed using the default settings of ShaEP [4] (commands #11 and 12). While this basic arrangement works well for COX-2, the yield can be improved with certain targets by either lowering or increasing the charge effect. The NIB screening success for Models I and II (Figure 4B,E) is estimated using the early enrichment and area under curve (AUC) values (Table 2 and Table 3) and the receiver operating characteristics (ROC) curves (Figure 4C,F) calculated with ROCKER [6] (commands #13 and 14). The compounds skipped during the ligand preparation or the NIB screening (Table 1), if any, are not considered in the AUC calculation. The Model II screening produced a higher AUC value than the flexible docking with the DUD set; however, the very early enrichment, or EFd 1%, was not better with either of the models using the OBABEL-generated conformers than what PLANTS produced (Table 3 versus Table 4). Notably, the enrichment was higher for both NIB models, surpassing the flexible docking yield, when the rigidly docked single/multiple conformers were generated using, for example, RDKit instead of OBABEL (Table 3; Figure 4C,F).

### 2.9. Feedback Loop—Fine-Tuning the Cavity Detection Settings

Following the rigid docking, one should inspect best-ranked docking poses and fine-tune the NIB models with the benefit of hindsight (Figure 1). For example, one can extract the 50 best-ranked docking poses (molpicking.bash; commands #15–20) and visualize the predicted binding modes using the preferred 3D viewer (e.g., BODIL) [5]. With the benchmark test sets, one can apply the trial-and-error approach, where the usefulness of each PANTHER setting generating the model (Figure 4B,E,H) can be assessed. This sort of training is not possible without verified active and inactive compounds or expert intuition to assess the effect of the applied changes. For COX-2, the DUD/DUD-E test sets [18,19] include both active ligands and inactive decoy molecules (Table 1) for assessing the fitness of the NIB models. The first-tried default settings frequently work well in the NIB screening [3] (e.g., Model I in Figure 4B,C); nevertheless, visualization of the models and docking poses and fine-tuning of the cavity detection settings is recommended (-ldlim 1.5 Å was used for Model II in Figure 4E,F). Better models might be acquired, for example, by varying the centroid position (Figure 4A), the cavity detection radius (Figure 4B), and the atomic radii of the protein residues (a PANTHER library rad.lib), the ligand distance limit (Figure 4E,F) or the filler atom packing method (Figure 4G).

The third NIB model, or Model III (Figure 4H), was generated using the otherwise same settings as Model II (Figure 4E), but the default face-centered cubic (FCC) packing was changed to the less dense body-centered cubic (BCC) method (command #21; Figure 4G). Model III can also be generated using an input file (final_panther.in in the Supplementary Material) instead of the elaborate terminal command. Though only the packing method was altered during the cavity-detection phase, the composition and shape of these two negative images differ markedly (Figure 4E versus 4H). The NIB screening/docking (command #22) and the enrichment analysis (command #23) indicate that Model III does not produce higher EFd 1% than the prior models when the single/multiple ligand 3D conformers were prepared using OBABEL (Table 2 and Table 3; Figure 4B,E). Yet again, the NIB screening results are considerably better for the third model, far surpassing the yield of PLANTS docking, especially, if the conformer generator RDKit was employed (Figure 4C,F versus 4I; Table 3 versus Table 4).

**Figure 6:**
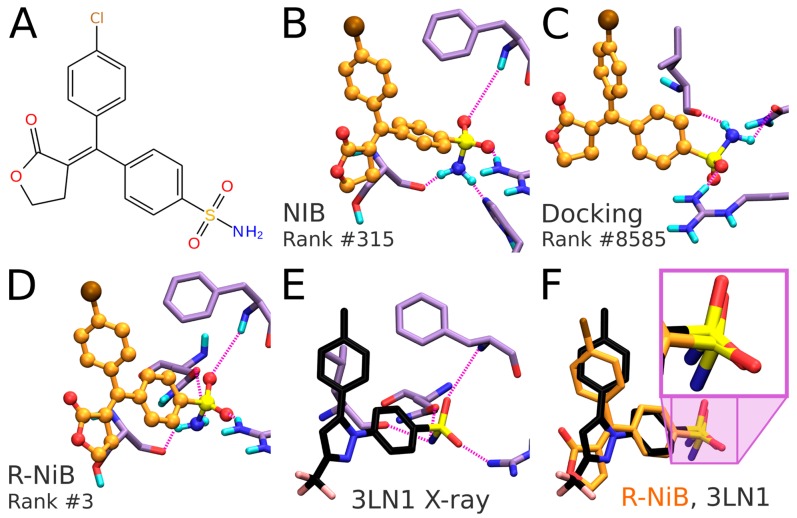
The predicted binding poses and ranking of a known inhibitor for comparison. (**A**) The 2D structure of an established cyclooxygenase-2 (COX-2) inhibitor ZINC03959950 included as an active ligand in the DUD test set [18]. (**B**) The negative image-based (NIB; Figure 1) [1] screening puts forth roughly similar pose for the inhibitor (yellow ball-and-stick model) as (**C**) the flexible docking with PLANTS [17] and (**D**) the negative image-based rescoring (R-NiB; Figure 2) [16]. However, the ranking of the inhibitor differs a lot between these approaches. In the top-ranked poses (**B**–**D**), the inhibitor’s sulfonamide group H-bonds (pink dotted lines) with the corresponding protein residues (purple stick models; protonated A chain of PDB-entry 1CX2 [20]) differently. (**E**) Notably, the verified binding mode of the inhibitor celecoxib (black stick model; PDB: 3LN1 [2]) indicates how the sulfonamide should be placed inside the cavity. (**F**) In fact, the comparison of celecoxib and the docked inhibitor (yellow stick model) binding modes shows that the sulfonamide placement (zoomed into in the magenta box) by the R-NiB is a match. The NIB selects highly similar alignment for the group as the R-NiB (**B** versus **C**) and the poorest choice is made by the default docking scoring of PLANTS (**E** versus **C**). The top-ranked inhibitor binding modes for both the NIB screening (conformers from the RDKit routine; Table 1) and rescoring were acquired using Model III (Figure 4H; PDB: 3LN1; Table 3).

These enrichment metrics (Table 2 and Table 3; Figure 4) indirectly suggest that the rigid docking with the ab initio generated ligand conformers is able to predict the binding poses of the COX-2 active ligands reasonably well. More to the point, the NIB, that ranks the ligands based on the cavity-based similarity, fare better than the ChemPLP scoring in PLANTS (Table 2 and Table 3). As with any docking methodology, it is prudent to compare the predicted poses to experimentally verified binding modes. Here, a close inspection is done for the best-ranked docking pose of an established COX-2 inhibitor included in the DUD set (Figure 6A,B). The overall alignment of the compound in question is roughly similar between the top-ranked NIB screening and the flexible docking poses (Figure 6B versus 6C,D), but, more importantly, the NIB proposes a binding pose resembling that of structurally related inhibitor celecoxib (Figure 6B versus 6E). As a result, the NIB screening provides a lot higher ranking for the inhibitor than the regular flexible docking (NIB rank #315 versus PLANTS rank #8585). The same ranking order, but to a lesser effect, is acquired for the celecoxib (NIB rank #125; PLANTS rank #295).

### 2.10. Negative Image-Based Rescoring of Flexible Docking Solutions

In addition to the cavity-based rigid docking (Figure 1 and Figure 4), the NIB methodology can be used to rescore existing docking solutions for improving the flexible molecular docking performance (Figure 2) [16]. The preliminary docking with the target protein can be performed using any flexible docking algorithm such as PLANTS [17] or AUTODOCK [30]. Multiple docking poses are outputted and, if need be, converted into the MOL2 format for the rescoring with the R-NiB. Next, the same cavity space used in the flexible docking is again used to generate a NIB model, which encompasses the key shape/charge features needed for the ligand binding, using PANTHER [3]. Finally, the flexible docking poses are compared against the cavity-based NIB model using ShaEP [4] without the time-consuming geometry optimization (--noOptimization) that is done during the rigid docking (Figure 3).

The COX-2 ligands [18,19] were docked flexibly using the default settings of PLANTS [17] after the ligand preparation with LIGPREP in MAESTRO in our previous study [16]. The used PLANTS input file and the explicit docking poses are included in the Supplementary Material. Models I–III (and alternative PDB-entry 1CX2-based Models IV–VI) generated for the NIB screening (commands #9, #10, and #21) were directly used with the R-NiB. Altogether, 10 alternative flexible docking poses for each ligand (Table 1) were rescored using ShaEP [4] without the geometry optimization (commands #24–26) using the --noopt option. The ShaEP result files containing multiple alternative poses for each rescored compound were trimmed to include only the best-ranked poses for the enrichment calculation (trim_shaep.bash; commands #27–29). The EFd 1%, EFd 5% and even the AUC values (commands #30–32) indicate that the R-NiB improved docking performance for the DUD set (Table 4). With the more demanding DUD-E set, the first model did not improve the enrichment, but the R-NiB was consistently able to improve the yield with the latter two models (Table 4).

In theory, the R-NiB or any other rescoring methodology can improve the docking yield (Table 4), because, while the original docking algorithm samples the “correct” ligand binding poses, its default scoring does not rate these specific poses high enough. Again, this effect is demonstrated by inspecting the predicted binding poses of an established COX-2 inhibitor included in the DUD set (Figure 6A). The R-NiB selects an alternative docking pose for the inhibitor (Figure 6D), which remains the best-ranked NIB screening pose (Figure 6B) regarding the sulfonamide group placement than the one ranked best by PLANTS (Figure 6C). Notably, the R-NiB ranking of the example compound is substantially higher than what PLANTS or the NIB suggested (R-NiB rank #3 versus NIB/PLANTS rank #315/#8585). By comparing this best-ranked inhibitor pose to structurally similar celecoxib bound at the COX-2 active site (Figure 6E; PDB: 3LN1 [2]), it is evident that it is spot on for the sulfonamide (magenta box in Figure 6F). Again, the R-NiB also provides a far better ranking for the inhibitor celecoxib than the flexible docking or NIB screening (R-NiB rank #42 versus NIB/PLANTS rank #125#295).

Despite this specific example (Figure 6), the ability of the R-NiB to improve docking performance could also be explained by the low scoring for the inactive decoys that align poorly with or further away from the designated cavity area. In any case, it is always recommended to extract a few of best-ranked docking poses (e.g., ~50–100) to see if the docking or rescoring results make sense at the atomistic level (molpicking.bash; commands #33–35).

### 2.11. Consensus Scoring: Balancing the Scoring Functions

**Figure 7:**
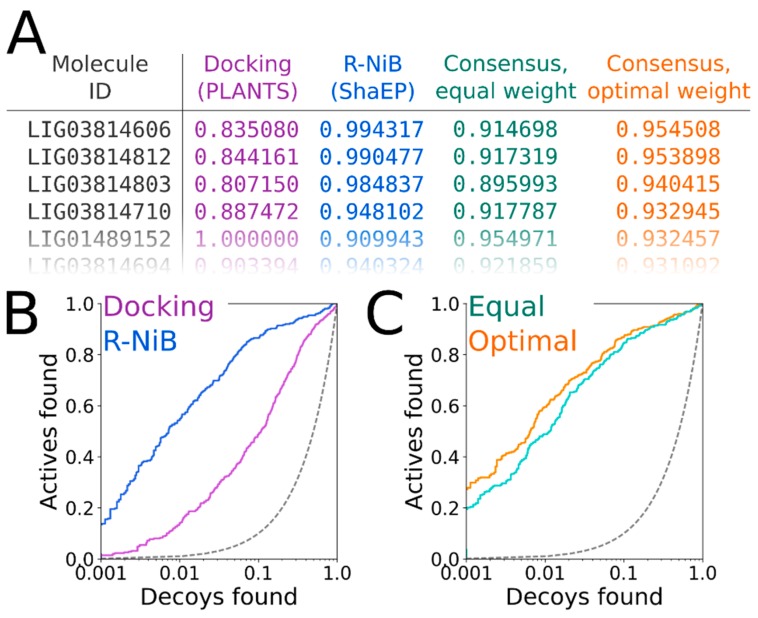
The consensus scoring of docking results using the negative image-based rescoring. (**A**) The highest PLANTS [17] scoring (magenta) and the ShaEP-based negative image-based rescoring (R-NiB) (blue) values for the docked DUD data set (both normalized from 0 to 1) are combined for the re-ranking of the compounds (green and orange). The aim was to improve the docking performance regarding the EFd 1% (Table 4) by applying either equal (weight = 0.50, green) or optimal weight (weight = 0.75, orange) on the scoring functions. The values are shown for the top-ranked molecules that were rescored using Model III (Figure 4H; Table 4). (**B**) Based on the semi-log10 scale (only *x*-axis logarithmic) receiver operating characteristics (ROC) curves, the straight-up R-NiB (weight = 1.00; Table 4; blue line) clearly outperforms the original docking scoring (magenta line; Table 4). (**C**) When a higher enrichment is sought via systematic re-weighting, the result is a consensus score with the optimal weight (orange line; Table 4) that produces higher early enrichment than the equal weight (green line; Table 4). Accordingly, relying more on the cavity-based ShaEP scoring than the original PLANTS docking or ChemPLP scoring produces a better yield. See Figure 1 for interpretation.

The ShaEP scoring can be combined with the original PLANTS scoring in the consensus scoring [16] (Figure 7A). The R-NiB scoring relies solely on the shape/electrostatics similarity calculated with ShaEP (weight of 1.00 in Table 4) and, accordingly, PLANTS is only responsible for sampling the ligand poses. Though the PLANTS docking enrichment is not as high as that of the best NIB or R-NiB results (Table 3 and Table 4), the flexible docking poses contain better matching geometries with the target’s cavity than what the ab initio conformer generators produce (Figure 6B versus 6D). However, the R-NiB performance can be improved in some cases by incorporating the original docking scoring to the ShaEP-based compound ranking. This requires the normalization of the scores outputted by both ShaEP and PLANTS, after which the weight of the two functions is balanced for the optimal effect (Figure 7A). In general (but not always), applying an equal weight (a weight of 0.50 in Table 4) between the scoring functions produces better enrichment than what the PLANTS docking or R-NiB produces alone; still, the optimal weight varies for different targets [16].

The normalization and re-weighting of the PLANTS and ShaEP scoring (Figure 7A) are performed using a BASH script (consensus.bash; commands #36–38), which is specific for the combined PLANTS and ShaEP usage. The consensus scoring for the DUD docking poses produced better EFd 1% values consistently if the optimal weight was systematically tested, whereas the equal weigh scoring (commands #39–41) improved on the R-NiB yield only with Model I (Table 4). With the DUD-E set, improvements over the R-NiB were consistent but also moderate with Models I and II (Table 4), but even a minor up-tick in the early enrichment could have substantial effects for real-life screening experiments. The early enrichment improvements over straight-up R-NiB and PLANTS docking (Figure 7B) through the consensus scoring are visible in the semi-logarithmic ROC curves of the DUD set with Model II (Figure 7C) when the optimal weigh was applied (Table 4).

## 3. Discussion

The cyclooxygenase 2 (COX-2) benchmarking example shows that the negative image-based (NIB; Figure 1) [1] screening or rigid docking is consistently producing higher enrichment than the regular flexible docking (Table 3 versus Table 4). The area under curve (AUC) values demonstrate the dominance of NIB over PLANTS [17] docking with both the DUD [19] and DUD-E [18] sets (Table 3). The NIB has been shown to outperform standard docking regarding the AUC values with a multitude of targets in prior studies [1,3,8]. On a practical level, the early enrichment values (calculated as true positive rates when 1 or 5 % of the decoys have been found) are a better measure of virtual screening success than the AUC values. Hence, it is noteworthy that the highest EFd 1% and EFd 5% values produced by the NIB screening with the COX-2 sets are also higher than those given by the PLANTS docking (Table 3 versus Table 4).

The better performance of the cavity-based rigid docking routine over the flexible docking suggests that the shape complementarity is a crucial factor for the COX-2 inhibitor binding, at least with the benchmark test sets. That is not to say that other docking algorithms or PLANTS cannot outperform the NIB methodology in virtual screening experiments on a case-by-case basis [3]. Both the composition of the test sets and the target’s cavity geometry are bound to lower the effectiveness of the cavity-based rigid docking in comparison to the flexible docking in some cases. Moreover, the COX-2 benchmarking (Table 2 and Table 3) indicate that the NIB model composition (Figure 4B,E,H), the input 3D protein structure conformation (Figure 4C), and the ligand conformer generation method (Table 1; Figure 4C,F,I) profoundly affect the rigid docking success (Table 3).

The NIB model generation is a straightforward process when the target protein’s ligand-binding site is a well-defined cavity (Figure 1 and Figure 4). The charge distribution and dimensions of the model can be improved using different PANTHER [3] options such as the cavity centroid (center(s), -cent; Figure 1 and Figure 4A), cavity detection radius (box radius, -brad; Figure 1, Figure 4A and Figure 5D), and filler atom packing (packing method, -pack; Figure 4G), as well as via alternative residue protonation (Figure 4B) or input structure conformation (PDB: 3LN1 versus 1CX2; Figure 4C; Table 3). A less direct approach might be needed when dealing with shallow surface pockets, as well as large or even open cavities without clear geometrical limits. To prevent the model protruding too far from the intended cavity area, one can limit the NIB model expansion using an already bound ligand (ligand distance limit, -ldlim; Figure 4D,E) or other selected residues (basic multipoint, -bmp) inside the cavity or even lining a groove on the protein surface. In addition, the NIB model can be forced to a certain subsection of the cavity by applying the multibox option (-mbox; Figure 5A), which allows the use of several coordinate points to describe a cavity of irregular or arbitrary shape.

Importantly, the COX-2 example shows that the rigid docking (Figure 1) is not only dependent on the NIB model shape/electrostatics but that the ligand 3D conformers have a substantial effect (Table 3). In the case of COX-2, the NIB screening works remarkably well when only a single low-energy 3D conformer is used in the rigid docking for each compound (Figure 4; Table 2). In fact, the results suggest that the costly use of multiple conformers (Figure 3; Table 3) could improve the NIB screening performance consistently with the DUD set only when the conformers were outputted by the RDKit (Table 3; Figure 4). With the more demanding DUD-E set, only the AUC values were improved consistently using the multiple conformers outputted by MARVIN or RDKit in the rigid docking (Table 3; Figure 4H,I).

Prior to this study, the ligand 3D conformer generation had been tested thoroughly for the purpose of assessing its effects on the ligand-based screening [31,32]; however, the benchmarking (Table 3) shows that the conformer composition is equally important for the NIB screening (Figure 1). OBABEL was used in the ligand preparation in the benchmarking example due to its ease of use and open source status (Table 1). The cavity-based rigid docking, especially using the single low-energy 3D conformers, produced consistently higher AUC values than the PLANTS flexible docking, regardless of the employed ligand preparation method (Table 3 versus Table 4). Nevertheless, the NIB screening done using the multiple ligand conformers outputted by OBABEL (Table 1) produced weaker enrichment than the other software such as RDKit (Figure 4C,F,I; Table 3). Though RDKit produced reasonable ligand conformations and excellent NIB screening results with the COX-2 test sets (Table 3), in practice, its successful usage requires basic knowledge of PYTHON programming and, generally, more effort than the other software (Table 1).

The effectiveness of the outputted 3D conformers differed between the software in the NIB screening. Their rigid docking performance is undoubtedly case-specific, and one should not draw too far-reaching conclusions based on the COX-2 benchmarking only. For example, the single low-energy conformers worked well with COX-2 (Figure 4; Table 2) likely due to the specific composition of the test sets and the flat or unfussy dimensions of the target cavity. The biologically relevant binding poses of the ligands, which are fundamentally sought after in the molecular docking are not necessarily close to the ab initio calculated energetic minima. Thus, the utilization of multiple alternative ligand 3D conformers in the cavity-based rigid docking should also improve the screening yield. The docking results vary significantly depending on the rotatable bond number of the molecules, the target protein’s ligand-binding cavity properties, and, above all, the selected non-default settings. Not all permutations could be tested for the ligand preparation, and it is fully possible that there exist better settings for the tested conformer generators (Table 1). Nonetheless, the results show that the composition or “quality” of the conformers (Table 2 and Table 3) is more important than their sheer quantity (Table 1) in the NIB screening.

The negative image-based rescoring (R-NiB; Figure 2) produces better enrichment than the original PLANTS docking (Table 4; Figure 7B) [16] or the standard NIB screening (Figure 1; Table 3) with the COX-2 test sets. The improvement is consistent with the DUD set, although the crudest or bulkiest NIB models did not improve the performance with the DUD-E set (Models I and IV in Table 4 and Figure 4). Accordingly, the R-NiB produced higher AUC values than the PLANTS docking with both the DUD and DUD-E sets using the alternative models (Table 4). In addition, the early enrichment improved over the docking with both test sets. The EFd 1% could be improved even further in comparison to the original docking if the scoring from both ShaEP and PLANTS was re-weighted (Figure 7A) for optimal performance in the consensus scoring (Table 4; Figure 7C).

The fact that R-NiB worked better than the NIB screening (Table 3 versus Table 4) is not surprising, because, during regular docking, the ligand conformers are sampled and optimized flexibly against the protein cavity. This structure-based sampling intrinsically affects the conformer composition and their placement against the protein’s cavity for the better (Figure 6). Paradoxically, a clear downside of the R-NiB (Figure 2) in comparison to the NIB screening (Figure 1) is the inflated computational cost of the flexible docking sampling prior to the cavity-based rescoring. The rescoring itself is ultrafast (Figure 3), as no geometry optimization between the template model or the ligand conformers is needed [16]. The user simply outputs several poses for each docked ligand (e.g., *n* = 10) to have enough explicit solutions to re-rank and improve the docking performance utilizing the cavity’s shape/charge information. The NIB screening (Figure 1) is faster than the regular docking precisely because the ligand conformers used in the rigid docking have been prepared in advance (Table 1), and these same ligand sets can be used without bias for all targets [1,3,8]. In contrast, molecular docking, which treats the ligands or even the protein itself flexibly, produces more tailored and target-specific binding modes.

Flexible docking algorithms have been shown to reproduce experimentally-derived ligand binding poses (see, e.g., [33]), although they might not recognize them in all cases (Figure 6). Despite the relatively high expense of these computations, one can realistically expect that even the most costly docking simulations and post-processing schemes will become plausible if computing performance continues to improve in the post-silicon era [34]. Thus, the biggest hurdle of structure-based drug discovery (besides acquiring the relevant protein 3D structures) is not necessarily the ligand pose sampling or the computational efficiency, but the inability of the default docking scoring functions to recognize the high-affinity binding poses and the potent compounds from the vast screening databases [16,33]. Consequently, the development of reliable scoring functions and easy-to-use rescoring methodologies such as the R-NiB (Figure 2) [16] is needed to supplement the existing docking software.

## 4. Methods

### 4.1. Ligand Preparation

The test sets, containing both active ligands (ligs in Table 1) and decoy molecules that are assumed inactive (decs in Table 1) for cyclooxygenase-2 (COX-2) were acquired from the DUD (a Directory of Useful Decoys) [19] and DUD-E (a database of useful (docking) decoys -enhanced) [18] databases. The compounds were acquired in the SMILES (simplified molecular-input line-entry system) format for the 3D conversion, adding of partial charges, protonation/tautomerization, and generation of multiple low-energy 3D conformers. To avoid bias [35], the DUD set was downloaded originally in the MOL2 format and translated to the SMILES format using either OBABEL [21] or STRUCTCONVERT in MAESTRO 2017–1 (Schrödinger, LLC, New York, NY, USA, 2017).

The ligand preparation was performed using five 3D conformer generation routines (Table 1) to study the effect of the conformer composition to the efficiency of the NIB methodology. Either a single low-energy conformer or a set of multiple conformers was generated for each compound.

1. OBABEL. Open Babel Package or OBABEL 2.4.0 [21] was used to convert ligands from the SMILES format into the SYBYL MOL2 format. The protonation was set to match pH 7.4, and partial charges from the Merck Molecular Force Field 94 (MMFF94) [22] were incorporated. The 3D ligand conformers were generated using CONFAB in OBABEL [21].
2. MAESTRO. The 3D conversion of the ligands (SMILES-to-MOL2) was performed using LIGPREP in MAESTRO. The protonation was set to match pH 7.4, and potential tautomers were created. The ligand 3D conformer generation was performed with CONFGEN in MAESTRO using an OPLS3 (Optimized Potential for Liquid Simulations) force field [36].
3. MARVIN. MOLCONVERT 17.6.0 in MARVIN (ChemAxon) was used for the ligand 3D conversion (SMILES-to-MOL2). CXCALC 17.6.0 in INSTANT JCHEM (ChemAxon) was used to protonate and create the potential tautomers at pH 7.4 and generate 3D conformers for the ligands. The number of conformers was scaled from 1 to 64 according to the rotatable bond number calculated with MayaChemTools [37]. The partial charges for the ligands were set using the MMFF94 in OBABEL.
4. RDKit. RDKit open-sourced cheminformatics was used in the ligand 3D conversion (SMILES-to-MOL2) and conformer generation. The protonation at pH 7.4 was prepared using MARVIN, and partial charges were added using OBABEL with the MMFF94 force field [22].
5. PLANTS. The flexible docking poses (Table 1), which were outputted by the molecular docking software PLANTS 1.2 [17], were taken from a prior study [16] for the R-NiB testing (Figure 2). The initial ligand preparation, including the protonation and tautomerization at pH 7.4, the incorporation of OPLS3 [36] partial charges, and the 3D conversion (SMILES-to-MOL2) was done using LIGPREP in MAESTRO. The outputted docking pose number was set to 10 for each compound (Table 1). Notably, the flexible docking skipped more decoys than the ab initio generators, which biases the screening results to some extent (Table 1).

### 4.2. Protein 3D Structure Editing

The X-ray crystal structure of COX-2 with the bound inhibitor celecoxib (PDB: 3LN1) [2] was acquired from the Protein Data Bank (PDB) [38,39]. This PDB-entry is given as the model structure for COX-2 in the DUD-E, and, thus, it is used here to make the comparison easy against prior benchmarking studies. The BODIL Molecular Modelling Environment [5] is recommended for the examination of the screening protocols due to tested compatibility with the input/output files (Figure 1 and Figure 2); however, other 3D viewer software should work as well. In the benchmarking example, the PDB-entry’s A chain was extracted, and its amino acid residues and ligands were protonated using REDUCE 3.13 [24]. The protocol was also tested using an alternative COX-2 structure with the bound inhibitor SC-558 (PDB: 1CX2 [20]; A chain).

### 4.3. Negative Image Generation

The negative image, or NIB model generation, was performed using the default settings of PANTHER 0.18.21 [3], if not otherwise specified. The freely downloadable version of PANTHER [3] is available at the website (www.medchem.fi/panther). A few of the previous default settings were amended for this study (example file also available online). The fundamental input options and updates to the packing method selection in the model generation with PANTHER (Figure 4 and Figure 5) are shortly explained below.

Center(s) (-cent) option designates the user-defined (*X Y Z*) center coordinate(s) for generating the NIB model. The centroid should be within a protein cavity that can accommodate the envisioned ligand binding (Figure 4A). If this centroid is defined at an unfavorable location or even outside the cavity of interest, the subsequent similarity screening with ShaEP [4] is likely to result in poor enrichment and/or wrong docking poses. In addition, the given centroid, together with the box radius (-brad) option (Figure 4A), defines where the packing of the filler atoms, constituting the negative image, begins.

Box radius (-brad) option is a key determinant of how the pocket is filled, together with the selected centroid (Figure 4A versus 4B). The packing of the filler atoms starts at the corner of the initial detection box, which is centered on the given centroid and has a vertex length of 2× box radius. Accordingly, an alternative box radius value can alter the lattice position used by the packing method and, ultimately, the reach of the resulting NIB model inside the cavity. The initial box is reduced into a sphere that, in turn, is defined by the box radius. In practice, the value for the box radius option should be set in a way that the model produces enrichment in the benchmarking (Figure 4C).

Keep (-keep)/Do not fill (-nofill) option is used to define a list of ligand residues with which the NIB model should not overlap. The area taken by these user-defined residues is excluded from the resulting NIB model. By default, some commonplace cofactors (FAD, NAP, NDP, NAI, NAD, and FDA) and water molecules (HOH and WAT) are listed for this option, but the list can be edited to include or exclude any residues of the input PDB file.

Multibox (-mbox) option makes it possible to generate a custom NIB model utilizing multiple close-by centroids. This option is especially useful when a single centroid is not enough to build a model that mimics the irregular shape of the target protein’s cavity. For example, the negative image of a peptide-binding site or groove at the protein surface can be built by picking several neighboring centroids and utilizing them together with the multibox option (Figure 5A).

Basic multipoint (-bmp) option can be used to generate the pocket center based on any kind of residue included in the input PDB file (e.g., Ser516 in Figure 5B). The basic multipoint overrides the center option. This option is useful especially when there is a ligand bound at the cavity of interest to provide the centroid coordinate.

Ligand distance limit (-ldlim) option can be used to restrict the volume that the NIB model occupies based on a bound ligand residue in the cavity of the input PDB file (Figure 4D,E). When this option is used, only those filler atoms within the given distance of the ligand atoms are preserved in the resulting NIB model. While this option is generally very useful, the peril of applying too short ligand distance is that the eventual model could mimic the existing bound ligand too closely (Figure 4D versus 4E), causing the early enrichment to fall (Figure 4C versus 4F).

Packing method (-pack) option, which is by default face-centered cubic (FCC), adds the filler atoms of the NIB model. The FCC has been altered to correspond the correct lattice in the updated version of PANTHER [3]. In addition, a body-centered cubic lattice (BCC) has now been implemented, and the cubic packing (CUBE) remains as the third option (Figure 4G). The FCC (0.74) has a higher packing fraction than the BBC (0.68; Figure 4B–E versus 4H) or the CUBE (0.52). While the FCC typically produces reasonable results, the BCC, for example, can produce better results depending on the cavity (Figure 4F versus 4I).

The input files and NIB models used in the benchmarking example (Figure 1 and Figure 2) are given in the Supplementary Material.

### 4.4. Cavity-Based Rigid Docking and Similarity Comparison

The NIB screening (Figure 1) and the docking rescoring (Figure 2) are done using a similarity comparison algorithm ShaEP 1.1.2.1036 [16]. Both the shape and electrostatics of the ligand conformers are compared against the template NIB model with an equal amount of weight (electrostatics = 0.5 versus shape = 0.5) to produce the best match. It should be noted that the early enrichment values and molecule rankings can vary ~1–2% between otherwise identical NIB screening runs. These minor discrepancies likely result from arbitrary features of the geometry optimization algorithm, and the results may vary more if different versions of ShaEP algorithm are used. In the R-NiB, several ligand poses (*n* = 10) outputted by the molecular docking software PLANTS [17] are not geometry optimized, i.e., docked rigidly (--noopt) in respect to the cavity-based template model, but they are only rescored using ShaEP [4].

### 4.5. Consensus Scoring

By combining the ShaEP-based R-NiB scoring with the PLANTS-based molecular docking scoring, it is possible to improve the flexible docking yield further (Figure 7). This involves the normalization of both the ChemPLP scoring of PLANTS and the ShaEP scoring into a matching range from 1 to 0 and the combining of the two sets of values to acquire a consensus score. The approach used here does not focus on the same ligand poses in the scoring process; instead, the best values produced by PLANTS and ShaEP for each screened compound (not necessarily the same conformer) are used. The weighting between the two scoring functions is done using a BASH script (consensus.bash) given in the Supplementary Material.

### 4.6. Figure and Table Preparation

Figure 1, Figure 2, Figure 3, Figure 4, Figure 5 and Figure 6 were prepared using BODIL [5], MOLSCRIPT 2.1.2 [40], RASTER3D 3.0.2 [41], and VMD 1.9.2 [42]. ROCKER 0.1.4 [6] was used to plot the receiver operating characteristics (ROC) curves with the semi-log10 scale (only *x*-axis logarithmic) and to calculate the early enrichment and area under curve (AUC) values in Table 2, Table 3 and Table 4. The enrichment factors were calculated as true positive rates when 1 or 5% of the decoys have been found (EFd 1% or EFd 5%), and the standard deviation of the AUC was acquired using the Wilcoxon statistic [6,43].

## 5. Conclusions

This study described the practical steps and software settings to be used during the negative image-based (NIB; Figure 1) screening or the negative image-based rescoring (R-NiB; Figure 2). Cyclooxygenase-2 (COX-2; Figure 1) was used as a benchmarking example for the NIB protocol from the ligand 3D conformer generation, the protein 3D structure preparation, and the NIB model generation to the similarity comparison or rigid docking (Figure 3). The input files, specific software settings, easy-to-use scripts, and terminal commands themselves were provided with thorough user guidance. The issues that arise from this practical screening example were detailed; moreover, other software settings relevant for the NIB implementation with different targets were discussed. A special focus was put on testing the applicability of different ligand conformer generation software for the NIB screening usage (Table 1, Table 2 and Table 3). Moreover, practical instructions were provided for the rescoring of flexible docking solutions output by the docking algorithm PLANTS using the R-NiB protocol (Figure 2). The R-NiB produces better enrichment than either the NIB screening or the flexible docking with the COX-2 example—a further boost is provided by the consensus scoring that combines the original docking scoring and the R-NiB scoring for optimal enrichment (Table 4; Figure 7). In summary, the study provides clear-cut instructions on how to perform rigid docking or docking rescoring with the NIB methodology using non-commercial software and a practical benchmarking example.

## Figures and Tables

**Table 1:** Ligand 3D conformers for the cyclooxygenase-2 benchmarking.

	Compounds		3D Conformer ab Initio Generation				Flexible Docking
	Class ^(1)^	SMILES ^2^	OBABEL ^3^	MARVIN ^3^	MAESTRO ^3^	RDKit ^3^	PLANTS ^4^
**License ^5^**	-	-	OS	AF	$$	OS	AF
DUD	ligs	348	3695	24,477	1650	12,850	4470
	skipped	-	1	0	0	1	0
	decs	12,464	620,660	695,202	89,218	329,301	118,440
	skipped	-	201	0	3	15	1818
DUD-E	ligs	435	9306	22,384	2322	16,069	4460
	skipped	-	17	1	0	0	0
	decs	23,144	1,053,413	2,405,040	212,014	807,066	247,600
	skipped	-	1335	20	8	13	8

^1^ The ligs refer to active compounds, and decs refer to inactive decoy compounds included in the DUD/database of useful (docking) decoys -enhanced (DUD-E) databases for the COX-2. The ligs/decs skipped refer to the total amount of molecules (not conformers) that were skipped either during the ligand preparation or rigid/flexible docking.^2^ The original compounds were included in the DUD/DUD-E as simplified molecular-input line-entry system (SMILES) strings before the 3D conversion. ^3^ The ligand 3D conformer numbers used by ShaEP from the 3D conformer generation software. ^4^ The ligand 3D conformers outputted by the docking software PLANTS [17] were acquired from a prior study [16]. The conformer number was set to 10 for each compound during the flexible molecular docking. ^5^ The ligand 3D conformer generators are divided roughly into three license categories: Commercial ($$), academic free (AF), and open source (OS). OBABEL has GNU or general public license. RDKit is under the Berkeley Software Distribution (BSD) license. MARVIN and MAESTRO are under copyright. Not applicable sections are marked (-).

**Table 2:** Negative image-based screening using the single low-energy conformers.

PDB Code	NIB Model ^1^	DUD				DUD-E			
		MAESTRO	OBABEL	MARVIN	RDKit	MAESTRO	OBABEL	MARVIN	RDKit
3LN1	Model I								
	AUC	0.82 ± 0.01	0.79 ± 0.01	0.65 ± 0.02	0.82 ± 0.01	0.62 ± 0.01	0.59 ± 0.01	0.65 ± 0.01	0.65 ± 0.01
	EFd 1%	10.1	11.8	5.7	12.7	0.0	0.9	0.5	0.2
	EFd 5%	42.8	17.0	29.9	35.7	0.7	1.4	5.3	3.2
	Model II								
	AUC	0.88 ± 0.01	0.86 ± 0.01	0.73 ± 0.02	0.88 ± 0.01	0.72 ± 0.01	0.70 ± 0.01	***0.73 ± 0.01***	***0.73 ± 0.01***
	EFd 1%	23.3	5.8	9.5	23.3	0.5	0.2	0.7	0.5
	EFd 5%	60.1	18.7	37.9	56.5	1.6	2.3	26.0	15.9
	Model III								
	AUC	0.88 ± 0.01	0.88 ± 0.01	0.65 ± 0.02	0.88 ± 0.01	***0.73 ± 0.01***	0.72 ± 0.01	***0.73 ± 0.01***	***0.73 ± 0.01***
	EFd 1%	***31.0***	7.5	16.4	24.8	4.1	0.7	***12.0***	7.1
	EFd 5%	58.0	44.4	34.5	58.8	27.4	21.5	***31.6***	28.5
1CX2	Model IV								
	AUC	0.83 ± 0.01	0.80 ± 0.01	0.78 ± 0.01	0.86 ± 0.01	0.64 ± 0.01	0.59 ± 0.01	0.64 ± 0.01	0.68 ± 0.01
	EFd 1%	15.8	0.9	10.6	15.3	0.0	0.2	0.5	0.0
	EFd 5%	41.7	26.2	34.5	44.1	2.1	2.3	5.8	4.1
	Model V								
	AUC	***0.89 ± 0.01***	0.85 ± 0.01	0.86 ± 0.01	***0.91 ± 0.01***	0.72 ± 0.01	0.68 ± 0.01	***0.74 ± 0.01***	***0.75 ± 0.01***
	EFd 1%	25.3	6.3	19.8	29.1	0.2	0.5	2.5	0.9
	EFd 5%	54.3	24.2	54.3	***61.4***	5.7	4.0	26.3	19.8
	Model VI								
	AUC	0.88 ± 0.01	0.87 ± 0.01	0.86 ± 0.01	***0.90 ± 0.01***	0.72 ± 0.01	0.70 ± 0.01	***0.74 ± 0.01***	***0.74 ± 0.01***
	EFd 1%	30.5	13.8	23.0	***31.1***	0.5	0.5	5.5	3.9
	EFd 5%	54.3	31.7	51.1	60.8	9.2	7.5	26.7	19.8

The AUC, EFd 1%, or EFd 5% values shown in bold and italics are the best scores of the DUD or DUD-E datasets within the error ranges. The scores that are higher than those produced by the multi-conformer NIB (Table 3) are underlined. ^1^ The NIB Models I–III and Models IV–VI were built using PDB-entries 3LN1 [2] and 1CX2 [20], respectively. The different PANTHER [3] settings are detailed in the Results section.

**Table 3:** Negative image-based screening using multiple ligand conformers.

PDB Code	NIB Model ^1^	DUD				DUD-E			
		MAESTRO	OBABEL	MARVIN	RDKit	MAESTRO	OBABEL	MARVIN	RDKit
3LN1	Model I								
	AUC	0.79 ± 0.01	0.73 ± 0.02	0.60 ± 0.02	0.84 ± 0.01	0.59 ± 0.01	0.56 ± 0.01	0.63 ± 0.01	0.64 ± 0.01
	EFd 1%	12.0	4.3	6.3	15.2	0.2	0.0	0.0	0.0
	EFd 5%	36.7	14.7	34.1	50.0	0.7	3.1	3.5	3.7
	Model II								
	AUC	0.87 ± 0.01	0.82 ± 0.01	0.64 ± 0.02	***0.90 ± 0.01***	0.69 ± 0.01	0.69 ± 0.01	***0.76 ± 0.01***	0.73 ± 0.01
	EFd 1%	15.5	0.0	0.6	35.1	0.0	0.7	0.2	0.5
	EFd 5%	53.9	3.5	41.3	69.5	0.7	3.1	24.7	15.9
	Model III								
	AUC	0.88 ± 0.01	0.80 ± 0.01	0.59 ± 0.02	***0.90 ± 0.01***	0.71 ± 0.01	0.69 ± 0.01	0.73 ± 0.01	0.73 ± 0.01
	EFd 1%	27.8	0.3	18.6	43.1	1.1	0.7	***11.8***	8.3
	EFd 5%	60.7	20.7	42.1	***79.9***	13.8	5.5	***39.1***	32.9
1CX2	Model IV								
	AUC	0.81 ± 0.01	0.69 ± 0.02	0.77 ± 0.01	0.85 ± 0.01	0.60 ± 0.01	0.54 ± 0.01	0.61 ± 0.01	0.64 ± 0.01
	EFd 1%	12.0	0.6	11.7	21.8	0.5	1.0	0.0	0.0
	EFd 5%	38.7	15.9	33.5	49.1	1.4	3.8	2.5	1.6
	Model V								
	AUC	0.89 ± 0.01	0.80 ± 0.01	0.88 ± 0.01	***0.91 ± 0.01***	0.71 ± 0.01	0.67 ± 0.01	***0.76 ± 0.01***	***0.76 ± 0.01***
	EFd 1%	22.1	0.0	14.9	40.2	0.0	0.5	0.2	0.0
	EFd 5%	59.3	11.0	49.9	70.1	0.5	2.4	24.7	15.4
	Model VI								
	AUC	0.88 ± 0.01	0.81 ± 0.01	0.85 ± 0.01	***0.91 ± 0.01***	0.69 ± 0.01	0.67 ± 0.01	0.74 ± 0.01	***0.75 ± 0.01***
	EFd 1%	23.5	1.7	15.5	***44.3***	0.2	0.2	1.8	0.9
	EFd 5%	57.0	10.4	45.6	75.9	2.5	2.6	24.2	20.9

The AUC, EFd 1%, or EFd 5% values shown in bold and italics are the best scores of the DUD or DUD-E datasets within the error ranges. The scores that are higher than those produced by the single-conformer NIB (Table 2) are underlined. ^1^ The NIB Models I–III and Models IV–VI were built using PDB-entries 3LN1 [2] and 1CX2 [20], respectively. The different PANTHER [3] settings are detailed in the Results section.

**Table 4:** Negative image-based rescoring and consensus scoring of docking results.

Screening Method ^(1)^	PDB Code	NIB Model ^(2)^	DUD				DUD-E			
			Weight ^(3)^	AUC	EFd 1%	EFd 5%	Weight ^(3)^	AUC	EFd 1%	EFd 5%
Docking	3LN1	-	-	0.81 ± 0.01	13.5	35.3	-	0.66 ± 0.01	5.7	21.6
R-NiB	3LN1	Model I	1.00	0.86 ± 0.01	20.1	48.3	1.00	0.63 ± 0.01	0.5	3.2
		Model II	1.00	***0.94 ± 0.01***	57.2	81.3	1.00	***0.78 ± 0.01***	11.3	30.0
		Model III	1.00	***0.94 ± 0.01***	54.3	79.3	1.00	***0.80 ± 0.01***	16.1	***37.7***
	1CX2	Model IV	1.00	0.86 ± 0.01	22.4	49.4	1.00	0.63 ± 0.01	0.5	3.2
		Model V	1.00	***0.94 ± 0.01***	64.9	***83.9***	1.00	***0.79 ± 0.01***	14.5	32.6
		Model VI	1.00	***0.94 ± 0.01***	58.9	77.0	1.00	0.77 ± 0.01	12.9	29.9
Consensus: Equal weight	3LN1	Model I	0.50	0.88 ± 0.01	29.0	55.5	0.50	0.66 ± 0.01	0.2	8.0
		Model II	0.50	***0.92 ± 0.01***	46.0	77.3	0.50	0.77 ± 0.01	13.8	32.4
		Model III	0.50	***0.92 ± 0.01***	48.9	75.9	0.50	***0.78 ± 0.01***	17.0	36.8
	1CX2	Model IV	0.50	0.87 ± 0.01	30.7	52.9	0.50	0.67 ± 0.01	0.2	10.1
		Model V	0.50	***0.93 ± 0.01***	56.9	77.0	0.50	0.77 ± 0.01	***18.4***	34.7
		Model VI	0.50	***0.92 ± 0.01***	51.7	74.7	0.50	0.76 ± 0.01	15.6	32.2
Consensus: Optimal weight	3LN1	Model I	0.60	0.88 ± 0.01	30.2	56.6	0.00	0.66 ± 0.01	5.7	21.6
		Model II	0.95	***0.94 ± 0.01***	58.3	81.6	0.55	0.77 ± 0.01	13.8	32.2
		Model III	0.75	***0.93 ± 0.01***	59.5	77.6	0.55	***0.79 ± 0.01***	17.7	36.8
	1CX2	Model IV	0.65	0.88 ± 0.01	33.0	53.7	0.05	0.67 ± 0.01	5.7	21.4
		Model V	0.85	***0.94 ± 0.01***	***65.8***	82.5	0.55	***0.78 ± 0.01***	***18.4***	35.4
		Model VI	0.85	***0.94 ± 0.01***	60.3	77.0	0.55	0.76 ± 0.01	16.3	32.0

The AUC, EFd 1%, or EFd 5% values shown in bold and italics are the best scores of the DUD or DUD-E datasets within the error ranges. ^1^ The COX-2 DUD/DUD-E test sets were docked originally in a prior study [16] using PLANTS [17]. The 10 outputted docking poses were used in the R-NiB or consensus scoring. ^2^ The NIB Models I–III and Models IV–VI were built using PDB-entries 3LN1 [2] and 1CX2 [20], respectively. The different PANTHER [3] settings are detailed in the Results section. ^3^ The R-NiB relies solely on the ShaEP scoring (weight = 1.00). The consensus scoring is done using the ShaEP scoring and the original docking scoring of PLANTS. The optimal weight between the two scoring methods was chosen based on the best EFd 1% enrichment for both the DUD and DUD-E test sets.

## Supplementary Materials

Supplementary materials can be found at https://www.mdpi.com/1422-0067/20/11/2779/s1.
